# Ex vivo Bone Models and Their Potential in Preclinical Evaluation

**DOI:** 10.1007/s11914-020-00649-5

**Published:** 2021-01-11

**Authors:** E. E. A. Cramer, K. Ito, S. Hofmann

**Affiliations:** Orthopaedic Biomechanics, Department of Biomedical Engineering and Institute of Complex Molecular Systems, Eindhoven University of Technology, P.O. Box 513, 5600 MB Eindhoven, the Netherlands

**Keywords:** Bone explants, Ex vivo bone model, Mechanical loading, Osteocytes, Bone formation, Bone resorption

## Abstract

**Purpose of Review:**

Novel therapies for damaged and diseased bone are being developed in a preclinical testing process consisting of in vitro cell experiments followed by in vivo animal studies. The in vitro results are often not representative of the results observed in vivo. This could be caused by the complexity of the natural bone environment that is missing in vitro*.* Ex vivo bone explant cultures provide a model in which cells are preserved in their native three-dimensional environment. Herein, it is aimed to review the current status of bone explant culture models in relation to their potential in complementing the preclinical evaluation process with specific attention paid to the incorporation of mechanical loading within ex vivo culture systems.

**Recent Findings:**

Bone explant cultures are often performed with physiologically less relevant bone, immature bone, and explants derived from rodents, which complicates translatability into clinical practice. Mature bone explants encounter difficulties with maintaining viability, especially in static culture. The integration of mechanical stimuli was able to extend the lifespan of explants and to induce new bone formation.

**Summary:**

Bone explant cultures provide unique platforms for bone research and mechanical loading was demonstrated to be an important component in achieving osteogenesis ex vivo. However, more research is needed to establish a representative, reliable, and reproducible bone explant culture system that includes both components of bone remodeling, i.e., formation and resorption, in order to bridge the gap between in vitro and in vivo research in preclinical testing.

## Introduction

Bone is a dynamic tissue that constantly adapts its architecture to the environment. This process is controlled by the interactive relationship between osteocytes, osteoblasts, and osteoclasts [1]. Osteoblasts are the bone-forming cells that deposit unmineralized matrix, called osteoid, while osteoclasts are responsible for resorption of bone matrix [2]. Osteocytes are embedded in the mineralized matrix and able to sense changes in their environment, which they transduce to other cells, including osteoclasts and osteoblasts [3].

With aging, the adaptive response is reduced, creating an imbalance between bone formation and resorption. This abnormal bone remodeling is a primary cause of bone diseases, including osteoporosis [4]. New anabolic and anti-catabolic therapies for osteoporosis are being developed and evaluated in preclinical testing processes, which depend heavily on animal models [5, 6]. In vivo experimentation is not only used to screen novel biochemical factors for metabolic bone diseases, it is also used to investigate bone development and growth, examine novel bone substitutes, and study impaired bone healing caused by clinical conditions, such as fracture non-unions, diabetes, and metastatic tumors [7–10]. Animal research is complex, expensive, time-consuming, and requires large sample numbers to show relevant effects [11, 12]. Therefore, in vivo testing is often preceded by in vitro studies as an initial evaluation of for example cytotoxicity, mechanism, and proliferative effect of novel therapies on bone cells [13, 14].

Since in vitro experiments are performed with standard culture vessels consisting of 2D single- or dual-cell cultures on tissue-culture plastic, they are simple and cost-effective. The complexity and relevance have advanced using 3D cell cultures on scaffolds or cell spheroids [15]. With excellent control over the culture conditions, such as cell number and differentiation, reproducible and high-throughput experiments can be generated [15]. However, the absence of the original extracellular matrix (ECM) and spatial arrangement as is seen in vivo can lead to changes in cell morphology and protein expression, predominantly in 2D cultures [6, 16•]. Consequently, the outcomes of in vitro experiments are often not representative of what is observed in vivo, which challenges the translation into clinical practice [15, 17].

Explant cultures, also known as organ or ex vivo cultures, maintain or grow explanted tissue in vitro, thus providing a unique platform to study cells in their native ECM with preservation of cell-cell and cell-matrix interactions as found in vivo. Moreover, ex vivo systems simplify the complexities of in vivo animal experiments, for example with the absence of systemic factors, thereby providing a controlled experimental setting where biological or mechanical factors can be examined independently [16•]. This could lead to valuable insights needed to bridge the gap between in vitro and in vivo experimentation, while it also addresses ethical considerations concerning animal testing by reducing and refining animal studies [13, 17, 18].

**Fig. 1:**
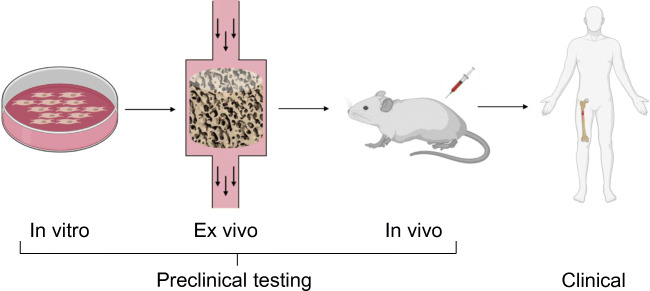
Traditionally, the development process for novel therapies related to bone starts with preclinical testing, consisting of in vitro experimentation and in vivo studies in animal models, followed by different phases of clinical trials in humans. Ex vivo models could potentially complement the pipeline of development when positioned between in vitro and in vivo testing. Image created with Biorender.com

Several explant models in bone research have been established and detailed descriptions of methodologies have been outlined [19]. The purpose of this article is to review the current status of the use of ex vivo culture systems and discuss challenges that need to be overcome in order to create representative, reliable, and reproducible model systems for bone explant culture. Special attention will be paid to the incorporation of mechanical loading as it is an essential factor in the bone environment [3]. Together, this will give insight into the potential of ex vivo bone models to become part of the preclinical testing process between simple in vitro testing and complex in vivo experimentation (Fig. 1).

## Mechanical Loading in Ex Vivo Bone Model Systems

Mechanical loading plays an important role in maintaining bone mass and can evoke changes in bone structure to better resist the loading [20]. Bone explant cultures are of great interest to investigate the effects of mechanical loading, because they provide a unique controlled environment to independently examine the influence of mechanical stimuli. Table 1 provides a detailed overview of studies involving mechanical loading in ex vivo bone cultures categorized by type of mechanical stimulus and how the mechanical loading influenced the outcome.

**Table 1:** Overview of studies involving mechanical loading in ex vivo bone cultures, listed by type of mechanical stimulus, species, origin of bone, type of explant, the presence of bone marrow experimental duration, and outcome (loaded vs. non-loaded samples)

Type of mechanical stimulus	Species	Origin of bone	Type of explant specimen	Bone marrow	Culture duration	Outcome	References
Perfusion	Rat	Femur	Bone shaft and condyles		14 days	Loading enhanced preservation of osteocyte viability	[21]
	Mouse	Femur	Whole femur	+	3 h	Ca^2+^ signaling of osteocytes in response to mechanical signals	[22]
	Chick	Calvaria (embryonic)	Rectangular segment		1 h	Fluid flow increased cell response rate observed from Ca^2+^ dynamics	[23]
	Human	Femur	Complete femoral head	+	12 h	Preservation of cell viability by perfusion through vasculature	[24]
Compression	Chick	Tibiotarsi (embryonic)	Bone shaft	−	2 days	Increase in G6PD expression and RNA synthesis in loaded samples	[25]
	Rat	Ulna	Bone shaft	−	24 h	PGI_2_, PGE_2_ and loading evoke an immediate release of G6PD	[26]
	Rat	Ulna, Calvaria	Bone shaft + rectangular segment	−	6 h	Release of PGI_2_ and PGE_2_, upregulation of G6PD upon loading in ulnae not in calvaria	[27]
	Rat	Metatarsal	Bone shaft	+	48 h	Greater osteocyte viability in loaded samples	[28]
	Mouse	Tibia	Whole tibia	+	48 h, 3 h	Intracellular Ca^2+^ oscillations in osteocytes in response to load and this diminished with age	[29], [30]
	Mouse	Fibula	Whole fibula	+	48 h	Osteocytes in larger lacunae responded stronger to loading	[31]
Perfusion + compression	Canine	Femur	Trabecular bone core	−	24 h, 6 h	Early response to loading by release of PGE_2_ and PGI_2_ and upregulation of G6PD	[32], [33]
	Bovine	(meta)tarsals	Trabecular bone core	−	28 days	Preservation of osteocyte viability in loaded samples, osteocyte-osteoblast communication visualized	[16•]
	Rabbit	Femur	Trabecular bone core	+	21 days	Osteoid formation, upregulation of osteogenic proteins and genes upon loading	[34]
*Zetos system*	Ovine, Bovine, Human	Femur, metacarpal, femur	Trabecular bone core	+	3 h	Optimized preparation for ovine, bovine and human trabecular bone cores	[35]
	Bovine	Sternum	Trabecular bone core	+	21 days	Elastic modulus increased when samples were loaded. Big ET1 + load increased bone formation	[36], [37]
	Bovine	Sternum	Trabecular bone core	−	18 days	Exogenously added sclerostin inhibited the increase in stiffness upon loading	[38]
	Bovine	Ulna, Sternum	Trabecular bone core	+	21 days	Increased osteoblast activity and osteogenic response in loaded groups	[39••]
	Bovine	Ulna	Trabecular bone core	+	26 days	Different intensities of loading resulted in different amounts of osteoid deposition	[40]
	Bovine	Ulna, Sternum	Trabecular bone core	+	21 days	Scanning acoustic microscopy can be used to assess changes in microelastic properties	[41]
	Ovine	Vertebra	Trabecular bone core	+	21 days	Shear stress in loaded samples was determined with computational modeling	[42••]
	Human	Femur	Trabecular bone core	+	14 days	TGFß3 + load enhanced osteocyte survival	[43]
	Human	Femur	Trabecular bone core	+	27 days	Increased preservation of osteocyte viability when loaded. Assessment of Zetos bioreactor for suitability of bone substitute testing.	[44•], [45], [46]
Perfusion + vibration	Ovine	Vertebra	Trabecular bone core	+	19 days	Mechanical loading influenced cilia expression of marrow cells	[47]
	Porcine	Vertebra	Trabecular bone core	+	19 days	Increase in bone formation upon stimulation of bone marrow	[48]
	Porcine	Vertebra	Trabecular bone core	+	28 days	Osteocytes were not responsible for increases in bone formation upon mechanical loading	[49]
Hydrostatic pressure	Bovine	Metacarpals	Trabecular bone core	−	22 days	Enhanced osteocyte viability and osteoid formation within loaded samples	[50]
	Chick	Femur (embryonic)	Whole femur	+	14 days	Increased mineralization within loaded samples	[51]
Stretch	Chick	Tibia	Bone slice		24 h	Increase in calcified area within loaded samples	[52]
Three-point bending	Rat	Femur	Whole femur	+	7 days	Mechanical stimulation resulted in increased stiffness	[53]
	Rat	Femur	Bone slice		8 days	Bone from aged rats showed diminished responses to loading	[54], [55]
Unloading	Rat	Tibia	Proximal epiphysis	+	28 days	Structural bone parameters and stiffness were decreased in unloaded samples	[56]

*ET1* endothelin-1, *G6PD* glucose-6-phosphate dehydrogenase, *PGE*_*2*_ prostaglandin E2, *PGI*_*2*_ prostacyclin, *TGFß3* transforming growth factor beta 3, **“**+” bone marrow present, “−“ bone marrow removed. No + or −, unclear whether bone marrow was present during culture

In the human body, bones experience differences in the type and level of mechanical stimuli, which include compression and perfusion induced strain. Various methods have been established to incorporate these different types of mechanical stimuli into bone explant culture systems. Perfusion systems were employed to generate load-induced strain leading to fluid flow within bone explants, thereby providing a mechanical stimulus with the additional advantage of improving nutrient delivery and waste removal [57]. Simple systems specifically designed to apply compressive forces to whole embryonic bones, which cause direct deformations of bone matrix, were used to characterize the early osteocyte’s response to loading [22, 25, 26, 57]. The combination of perfusion and compression, which is of greater physiological relevance for explants of weight-bearing bones, was included in advanced culture systems and allowed longer ex vivo culture periods [16, 32]. The combination of mechanical stimuli was further advanced into a platform, called Zetos, able to apply compressive forces onto trabecular bone cores with high precision under constant perfusion [58]. The compressive forces could be applied in a variety of different wave forms and frequencies to simulate walking or jumping for example, while also having the ability to measure mechanical properties, such as stiffness, in real time [58]. A different type of bioreactor system combined perfusion with low magnitude, high-frequency vibrations to evoke shear stresses within bone explants without associated deformations in the bone matrix [47, 48]. The absence of direct compression on the bone matrix was also realized in an ex vivo system utilizing cyclic hydrostatic pressure where gas surrounding the samples was compressed [50, 51]. Other, physiologically less relevant types of mechanical loading investigated in bone explant culture settings included static stretch and three-point bending [52–55].

### Influence of Mechanical Loading on Osteocytes in Bone Explants

To study osteocyte function in their native environment without the influence of other cells, bone marrow, surface cells, and other soft tissue cells can be removed from bone explants [16, 19, 59–61]. The preservation of an intact lacunar-canalicular network allows for maintenance of mature osteocytes, which is a great advantage over current tissue engineered in vitro systems where this advanced osteocyte network is not yet achieved. This osteocyte model was used in static culture to study the effect of biochemical factors on osteocytes as well as to analyze the interaction between osteocytes and other cell types that were exogenously seeded [16, 50, 62–65]. However, mechanical loading appears to be an important factor for culture of marrow deprived bone explants, probably because of the role of osteocytes as mechanosensors of bone. Through culturing bone explants in perfusion bioreactors, osteocytes retained viability and function for 14 days, whereas most of the osteocytes died or disappeared from the lacunae in static culture with viable cells only found on the edges because they could be reached by diffusion [16, 21]. Also, the application of either cyclic hydrostatic pressure or compressive loading showed to enhance osteocyte viability [28, 50]. When investigating the osteocyte response to mechanical loading, it was observed that the combination of perfusion and compression evoked an immediate release of prostaglandin E2 (PGE_2_) and prostacyclin (PGI_2_) followed by an upregulation of G6PD activity [32, 33]. The upregulation of PGI_2_ was not observed when non-load-bearing calvarial tissue was subjected to loading [27]. More recent research reported that the early release of PGE_2_ by osteocytes was related to increased levels of bone formation after 4 weeks of culture when osteoblasts were seeded onto bone specimens [16, 66]. Ex vivo bone cultures were further used to investigate another early event in the cascade of mechanotransduction, a peak in intracellular calcium (Ca^2+^). The culture system was designed to visualize, in real-time, Ca^2+^ dynamics in osteocytes in response to compressive loads [29, 30]. Building on this work, a setting was created in which the effects of fluid flow on osteocytes could be studied in the absence of mechanical deformation of the matrix by directly applying fluid flow into the marrow cavity [22]. Upon mechanical stimulation through fluid flow, osteocytes displayed upregulation of the Ca^2+^ response [22, 23]. Overall, the incorporation of mechanical loading into bone explant culture systems led to osteocyte responses, including increased osteocyte survival, PGE_2_ and PGI_2_ release, and Ca^2+^ dynamics, previously reported for in vivo and in vitro systems.

### Influence of Mechanical Loading on Bone Formation in Bone Explants

Mechanotransduction includes osteocyte signaling to other bone cells, such as osteoblasts and osteoclasts, thereby coordinating matrix remodeling [29]. Instead of flushing out bone marrow to study osteocytes exclusively, bone marrow can be preserved to investigate bone formation and resorption processes [36, 38]. The maintenance of bone marrow provides cellular heterogeneity, including osteoclasts, osteoblasts, and their progenitors, necessary for bone remodeling [47]. The contributing role of marrow in the bone remodeling process was demonstrated within trabecular bone cores using an ex vivo perfusion system combined with vibrational stimuli [48]. A positive correlation between induced shear stress in the bone marrow and bone formation was found [48]. Since osteocytes were unaffected by this type of mechanical stimulation, the increased bone formation indicated that marrow cells are also mechanosensitive and play a role in bone remodeling [49]. Moreover, osteocytes are known to affect bone remodeling by expressing a range of proteins and cytokines [67], [68]. For bone explants in static cultures, presence of signaling molecules included in the bone formation and resorption pathways, such as sclerostin and RANKL, was demonstrated [68–71]. However, the influence of mechanical loading on the expression of these proteins and whether they have a direct effect on bone remodeling in bone explant cultures has to be investigated.

Using the Zetos bioreactor system, trabecular bone cores showed increased preservation of osteocyte viability and increased amounts of bone formation observed from upregulated osteoblast activity, osteoid deposition, trabecular thickening, and a higher Young’s modulus, compared with unloaded samples [38–40, 44]. Analysis at multiple depths of the bone core displayed osteocyte viability throughout the thickness, suggesting that the combination of continuous perfusion and daily loading induced a fluid distribution that was able to reach the center of the bone explants [44•]. Survival of osteocytes in the core centers was further enhanced by the administration of TGFß3 [43]. Using the ability of the system to determine stiffness of the bone samples demonstrated that stiffness increased over time for samples subjected to daily loading [36, 40]. The increased stiffness was found to be dependent on the magnitude of peak strain and related to osteoid thickness [40]. The Zetos system measured stiffness of the total sample, whereas scanning acoustic microscopy was utilized in a similar ex vivo setup to map spatial variations of acoustic impedance, which is related to tissue stiffness, within single trabeculae [41].

The incorporation of mechanical loading, especially the combination of perfusion with another type of loading, has shown to be able to extend culture periods (up to 4 weeks) and was associated with stronger osteogenic responses compared with static cultures [16•, 36, 39••, 44••, 49]. This underlines the importance of bioreactors in addressing one of the challenges in bone explant culture, the limited lifespan [15]. Moreover, osteogenic responses of bone explants to biochemical factors were different when mechanical stimulation was included [26, 37, 43]. Therefore, the incorporation of mechanical stimuli is strongly suggested when examining the effects of biomaterials and drugs on bone explants, especially for long-term culture systems.

### Influence of Mechanical Loading on Bone Resorption in Bone Explants

Mechanical stimuli are essential factors in the bone remodeling process which involves not only bone formation but also resorption [3]. One of the rare studies that investigated osteoclast activity and resorption upon mechanical loading demonstrated the presence of osteoclasts, no active resorption, and no response to mechanical loading [39••]. However, the osteoclasts were still reactive when stimulated by retinoic acid. Bone resorption is known to increase in situations where mechanical loading is reduced or completely absent [3]. A constantly rotating bioreactor allowed culture of bone explants in an environment achieving near-weightlessness [56, 72]. After a 3–4-week culture, reduced levels of bone volume and mechanical properties were observed suggesting an increased resorption, although thorough analysis of osteoclast activity was not performed [56].

Up until now, studies involving mechanical loading in bone explant cultures showed a clear focus on achieving bone formation and neglecting osteoclast activity and resorption while both are key elements of bone remodeling in vivo. Consequently, culture medium is often supplemented with factors that stimulate osteoblasts in producing bone matrix, while osteoclasts are not stimulated and even might be suppressed. For static explant cultures, it was demonstrated that osteoclastogenesis could be induced by the bacterial factor LPS, parathyroid hormone, or pro-inflammatory cytokines, such as RANKL, MCSF, IL-6, and TNF-α [73, 74]. Therefore, more research is needed to provide osteoclasts with an environment that facilitates resorption within mechanically stimulated bone explant models in order to generate a model that comprises both elements of bone remodeling.

## Physiological Relevance of Bone Used in Explant Models

### Immature Vs. Mature Bone

Embryonic or neonatal bone is frequently used for bone explant cultures because it allows for long-term culture with cell viability being maintained for up to several months [75–77]. However, incomplete mineralization and lack of mature immune cells make immature bone not representative for clinical situations which most often are targeted by therapies [78]. The use of mature bone is recommended as a more relevant scenario in preclinical testing. However, its culture is challenging because the diffusion of nutrients is hampered by the thick calcified tissue and the fatty bone marrow, which causes cell death and matrix degradation in static cultures [79, 80].

### Animal Bone Vs. Human Bone

Therapeutics are predominantly developed for human applications, but bone tissue used in ex vivo testing often originates from animals, including rodents, canine, and bovine. Consequently, species-specific differences in macrostructure, microarchitecture, composition, and bone remodeling result in a lack of correlation between animal and human experimental outcomes [7, 81]. Rodent models for example, often used for ex vivo experimentation, have differences in bone composition, mechanical properties, and are missing the Haversian remodeling system, leading to a gap in translation of therapies from mice to human [6, 81, 82]. The use of bone tissue from larger animals is clinically more relevant with respect to size and with a similar lamellar structure relative to human bone [7]. In addition, bone explant material from large animals is usually obtained from slaughterhouse’s left-over material, such as ovine femora and bovine metacarpals, thereby circumventing the complicated regulations for animal studies and extra costs for animal care [35]. However, there is no animal model with an identical bone structure and physiology [7, 82]. Hence, responses to drugs, such as BMP-2 and bisphosphonates, demonstrated to be different in animals compared with humans [6, 14, 83].

Research involving human bone can be performed using left-over material from autografting procedures [61, 63, 68, 69, 84, 85]. Usually, these include small thin fragments (Ø = 1 mm) obtained from non-load bearing bones and do not include the extensive structure of trabeculae [61]. This is an important point of consideration as non-load bearing flat bones, such as calvariae, show a different osteogenic response to mechanical loading compared with trabecular bone from long bones [27, 39••, 41]. The use of human bone cores in ex vivo culture models could remove obstacles experienced in translating outcomes from animal experimentation to human applications. Trabecular bone cores (Ø = 6–20 mm, height = 4–7 mm), obtained from femoral heads or tibia plateaus removed during replacement surgeries, demonstrated successful preservation of viability ex vivo*,* when cultured up to 4 weeks [14, 35, 43, 44•, 46, 80, 86, 87]. Notably, a recent study reported that an entire human femoral head could be kept viable for 12 h when culture medium was perfused through the remaining vasculature [24].

Taken together, human bone tissue would be ideal for ex vivo cultures in terms of physiological relevance, but it has major downsides regarding availability and variability. This hampers large scale testing, which is needed to reach statistical significance in preclinical experiments. Therefore, benefit could be taken from easier accessible large animal bone specimens, usually obtained from slaughterhouse material, to create high throughput experimentation in order to establish reproducible systems.

### Ex Vivo Models for Bone Diseases

The use of human bone tissue is further complicated by the presence of underlying diseases and the difficulty to obtain healthy tissue to serve as control. Frequently, femoral heads originate from osteoporotic patients which might be on bone affecting medication. However, these diseased bones could also provide an opportunity to create a physiologically relevant osteoporotic explant model, which could be of great interest for preclinical drug testing. Also, research related to rheumatoid arthritis (RA), an inflammatory disease associated with bone destruction, could benefit from ex vivo human bone models when using small pieces of bone isolated from joints of RA patients [61, 68, 88, 89]. Cancer metastasis in bone could be simulated by co-culture of human bone fragments and human prostate or breast cancer cells leading to infiltration of malignant cells into the bone marrow cavities reflecting the bone metastatic niche [90–93]. Osteoarthritis is a disease that is characterized by cartilage degeneration as well as changes in the subchondral bone with a close interrelationship between osteoblasts, osteoclasts, and chondrocytes [94]. Therefore, a clinically relevant preclinical model for osteoarthritis would involve explant tissue consisting of articular cartilage and subchondral bone, the so-called osteochondral unit, which can be obtained from distal femoral condyles or femoral heads [95–97]. However, osteochondral cultures were primarily optimized to investigate cartilage regeneration without considering the optimal culture conditions for bone [95, 97, 98]. Recently, a culture platform with two separated media compartments was established, which achieved a better reproduction of the in vivo situation because signaling between the cartilage and bone could only happen through the subchondral bone plate [99].

## Correlation to In Vivo Data

In order to implement bone explant cultures as valuable platforms in preclinical testing, it would be desirable to have ex vivo osteogenic responses predictive of in vivo success. To examine the correlation between ex vivo outcomes and in vivo results, a multicenter analysis would be needed, such as the study of Hulsart-Billström and colleagues where in vitro and in vivo data of biomaterials for bone regeneration were compared and correlated using a scoring system [17]. Once enough data is available, such a thorough analysis should also be performed to investigate the correlation between ex vivo and in vivo studies.

A major issue in comparing and correlating ex vivo and in vivo outcomes is the typical length of the culture periods. Explant cultures have a duration of days to a few weeks associated with minor amounts of bone formation and resorption, while in vivo models take up several weeks to complete in small animals and multiple months for large animals accomplishing substantial changes [100, 101]. To overcome this issue, it would be ideal to adapt the different culture periods by also having in vivo results at early timepoints. Relating outcomes to in vivo results is further complicated by the variation in analysis techniques used, impeding direct comparison because bone responses are evaluated at different levels [101].

A few articles reported results of ex vivo research followed by in vivo evaluation intending to relate the outcomes [70, 102, 103]. However, different types of bones, calvariae and femur, were used and analysis was only performed on mRNA and protein level. Another study demonstrated increased amounts of bone formation in mouse calvariae culture upon administration of an anabolic small molecule [104]. Consistent with the ex vivo findings, increased bone formation was observed in a defect in vivo [104]. However, care needs to be taken with interpreting comparable outcomes as mechanisms behind the observed effects could differ, because parameters present in vivo are missing ex vivo, such as mechanical loading, the immune system, and an inflammatory response. These are known to be involved in bone regeneration. In addition, the required dosage of biochemical factors administered might require attention as uptake and transport differ between ex vivo and in vivo situations because the vascular network is missing. Hence, it cannot be expected that ex vivo cultures represent all processes happening in vivo and it needs to be evaluated whether ex vivo systems are complex enough to represent certain in vivo processes before they can be utilized as preclinical testing models.

## Applications of Ex Vivo Models in Preclinical Testing

With the preservation of cells in their native environment, bone explant cultures might be of added value for preclinical evaluation of novel therapies targeting diseased or damaged bone. To evaluate biomaterials, ex vivo bone defect models were established that comprised embryonic bones with a central segmental defect [105–108] and trabecular bone cores of mature bone with a defect created centrally [46, 86, 109]. Outcomes were limited to demonstrating osteocyte viability and cellular ingrowth from the bone into the material [86, 109]. Only one paper analyzed the osteogenic response in more depth by monitoring factors as Ca^2+^, ALP, and osteocalcin in the medium and showing new bone formation by incorporation of tetracycline which was released from the bone substitute [46]. These studies showed the first step in creating an ex vivo defect model, but further development is needed before standardized testing of bone substitutes can be achieved. Hence, an important component in fracture healing that is challenging to integrate ex vivo is the inflammatory response and associated hematoma formation because vascularization is lacking. This challenge was partly addressed by an ex vivo study utilizing a femoral defect model to implant hydrogels that released platelet-rich plasma, which contains many cytokines involved in bone regeneration and vascularization processes [110]. A different approach to address the vascularization problem included culturing of bone explants on the chick chorioallantoic membrane (CAM) [14, 107]. Invasion of human bone cores by CAM capillaries was realized and ECM deposition and mineralization was shown, although mainly caused by the infiltrated avian cells [14, 111].

For testing the potency of growth factors and small molecules for bone-related diseases, explants obtained from mouse calvarial bones are regularly used because it is a relatively simple and inexpensive model [62, 70, 78, 104, 112–124]. The addition of human prostate or breast cancer cells to mouse calvariae allowed investigating the effects of several potential cancer therapeutics [74, 125–133]. Physiologically more relevant bone explants derived from large animals or humans were seldomly used to investigate effects of exogenously added factors [37, 38, 84, 134].

## Challenges in Isolation, Culture, and Analysis of Bone Explants

Currently, bone explant cultures can take up months to complete and are performed manually creating operator-dependent variability [6, 78]. This leaves room for improvements on optimization of isolation procedures, standardization of culture conditions, and automation of analysis techniques.

### Preparation of Bone Explants

When tissue is harvested, it suffers from hypoxia and mechanical stress leading to cell death and changes in cell behavior as well as formation of bone debris clumps as a direct result of drilling [35, 86]. Consequently, an adaptation period characterized by an increased release of intracellular enzymes into the culture medium was observed during the first 2–6 days of culture [86, 135, 136]. Optimization of isolation could focus on reducing stress on cells during harvesting which could possibly shorten or refine the adaptation period.

### Culture of Bone Explants

No consensus is reached about which medium is optimal for which type of bone explant. This leads to a variety of different media supplemented with all kinds of factors, including bovine albumin, ascorbic acid, fetal calf serum, and dexamethasone [82]. Dexamethasone, frequently used for in vitro culture of osteogenic cells, showed to negatively affect osteocytes in bone explants [62, 64]. Also, the use of serum supplementation is debatable, because it has batch-to-batch variation leading to a reduced reproducibility, but its presence has shown to extend culture periods significantly [19]. Moreover, supplementation of medium adds charged molecules to the culture which can lead to disturbances of streaming potentials in the lacuna-canalicular network [137]. Serum alternatives, for example chemically defined serum-free media or human platelet lysate, are recommended to be tested for bone explant culture to overcome the issues with serum supplementation [138]. Overall, more research is needed to determine the optimal nutritional requirements for bone explants which might lead to enhanced cell activities and an increased lifespan.

Improvements can also be made on bioreactor design and usage, in order to achieve large-scale ex vivo experimentation. Most of the bioreactors are custom-built devices developed to answer a specific research question and only allow accommodation of bone explants with a specific shape and size. Only the Zetos platform showed to a certain extent the potency for screening factors and biomaterials under physiologically relevant loading [35, 58]. Furthermore, studies examining effects of different load intensities, waveforms, frequencies, or number of cycles are limited but needed to determine the ideal loading regime for bone explants to evoke an osteogenic response similar to in vivo.

### Analysis of Bone Explants

Whole bone explants pose challenges in the application of standard evaluation techniques because of the calcified bone and the fatty marrow limits dye penetration into the tissue [80]. Therefore, evaluation is often limited to measurement of markers, such as LDH, ALP, and TRAP, in the medium, which only provides insight into total turnover. In order to obtain local information, techniques need to be adapted and optimized, for example by longer incubation times and lowering temperatures to decrease metabolic activity of cells during viability analysis [80].

Histology in the form of H&E staining is generally performed to visualize bone matrix. However, decalcification of mature bone tissue is required, which complicates discrimination between old and newly formed matrix. The use of plastic embedding and advanced histological stains, such as Masson Golder stain, avoids decalcification and could be of interest to implement as standard in analysis of bone formation [35]. Where histological analyses show the status of bone at a specific point when the sample is sacrificed, techniques to evaluate bone formation over a period are preferred to reduce the needed sample size. Dynamic histomorphometry, which has already been used in ex vivo systems, allows for visualization and quantification of bone formation during culture by the administration of calcium binding fluorochromes at specific timepoints [44•].

Micro computed tomography (μCT) imaging is integrated in general bone research but only occasionally in explant cultures. This technique has a high potential for bone explant cultures as was shown with the visualization of remodeling [34, 48, 139, 140]. In addition, the combination of μCT and finite element modeling allowed quantification of local matrix strains and marrow stresses, used to demonstrate that values were in the range known to induce an osteogenic response [42, 48]. The use of computational models to simulate processes inside the bone under specific conditions could further assist in the interpretation of experimental outcomes.

Depending slightly on the research question, research involving bone formation or resorption within bone explants might benefit from a standardized analysis protocol that combines different evaluation techniques to get information about different stages of culture. This should include analysis of biomarkers in the medium as an indication of total tissue activity and histology, dynamic histomorphometry, and μCT imaging for local analysis. Moreover, these techniques are also used for animal testing and would therefore allow for easier correlation to in vivo outcomes [141, 142].

## Conclusion

A representative, reliable, and reproducible ex vivo system to assess the potency of novel treatments for damaged or diseased bone has yet to be established. The creation of standardized advanced models is hampered by different drawbacks of bone explant cultures, including limited lifespan in static culture and the absence of osteoclast activity and resorption, especially for mechanically loaded explants. To overcome these challenges, future research should focus on the incorporation of mechanical loading through standardized bioreactors and finding optimal culture conditions to allow osteoblasts as well as osteoclasts to fulfill their actions within the remodeling process. Furthermore, cultures with physiologically relevant bone tissue, ideally from large animals or humans, with optimized procedures of isolation, culture, and analysis are needed to establish platforms that could complement the process of preclinical testing. Thanks to the preservation of physical and spatial complexity, bone explant cultures could improve translatability between in vitro and in vivo studies and favors our ethical responsibility to reduce, refine, and replace animal testing.

## Data Availability

Not applicable.
